# Anti-inflammatory and anti-arthritic activities of ethanolic extract of *Myxopyrum serratulum* A.W. Hill

**DOI:** 10.1186/s42826-024-00220-8

**Published:** 2024-09-26

**Authors:** Sheela Rani T, Srikanth Jeyabalan, Sivaraman Dhanasekaran, Mahendran Sekar, Vetriselvan Subramaniyan, Ling Shing Wong

**Affiliations:** 1 Department of Pharmaceutical Chemistry, Sri Ramachandra Faculty of Pharmacy, Sri Ramachandra Institute of Higher Education and Research (DU), Chennai, India; 2 Department of Pharmacology, Sri Ramachandra Faculty of Pharmacy, Sri Ramachandra Institute of Higher Education and Research (DU), Chennai, India; 3 Pandit Deendayal Energy University, Gandhinagar, Gujarat 382007 India; 4 School of Pharmacy, Monash University Malaysia, Bandar Sunway, Subang Jaya 47500, Selangor, Malaysia; 5 Jeffrey Cheah School of Medicine and Health Sciences, Monash University Malaysia, Bandar Sunway, Subang Jaya 47500, Selangor, Malaysia; 6 Faculty of Health and Life Sciences, INTI International University, Nilai, 71800 Malaysia; 7 Department of Medical Sciences, Sunway University, Bandar Sunway, Subang Jaya, 47500 Malaysia

**Keywords:** *Myxopyrum serratulum*, Anti-inflammatory, Anti-arthritis, Cathepsin-D, Human health

## Abstract

**Background:**

Rheumatoid arthritis (RA) is a debilitating inflammatory disorder characterized by an overactive immune system targeting joints, leading to inflammation and intense pain. While current RA therapies effectively alleviate symptoms, they are often associated with significant side effects. This study aimed to assess the anti-inflammatory and anti-arthritic properties of an Ethanolic Extract of *Myxopyrum serratulum* A.W. Hill (EEMS) using animal models.

**Results:**

The acute toxicity study with EEMS (2000 mg/kg, p.o.) on rats showed no toxicity or mortality up to the highest dose. Inflammation was induced using carrageenan, and rats were treated with varying doses of EEMS (100, 200, and 400 mg/kg, p.o.) and diclofenac to assess anti-inflammatory effects. Anti-arthritic efficacy was evaluated using Complete Freund’s adjuvant (CFA)-induced inflammation, comparing EEMS to methotrexate. The results revealed dose-dependent anti-inflammatory effects of EEMS and a reversal of arthritic-induced weight loss in treated groups. Paw volume reduction was significant in both EEMS and methotrexate groups. Biochemical analyses showed elevated markers in the arthritic control group, which were normalized by EEMS and methotrexate. Notably, EEMS (400 mg/kg) significantly reduced cathepsin-D levels compared to the positive control. EEMS administration also lowered hepatic lipid peroxidation and increased endogenous antioxidants (SOD, GSH, and GPX). The 200 and 400 mg/kg doses reduced the iNOS/GADPH ratio, while the 400 mg/kg dose restored cellular and joint structure and significantly decreased IL1 levels.

**Conclusions:**

In conclusion, EEMS demonstrated substantial protective effects, mitigating health risks associated with chronic inflammation such as arthritis. These findings underscore the ethnomedical potential of Myxopyrum serratulum as a promising anti-inflammatory and anti-arthritis agent. The study suggests that EEMS could be a viable alternative or complementary therapy for RA, offering therapeutic benefits with potentially fewer side effects than current treatments.

## Background

**Fig. 1:**
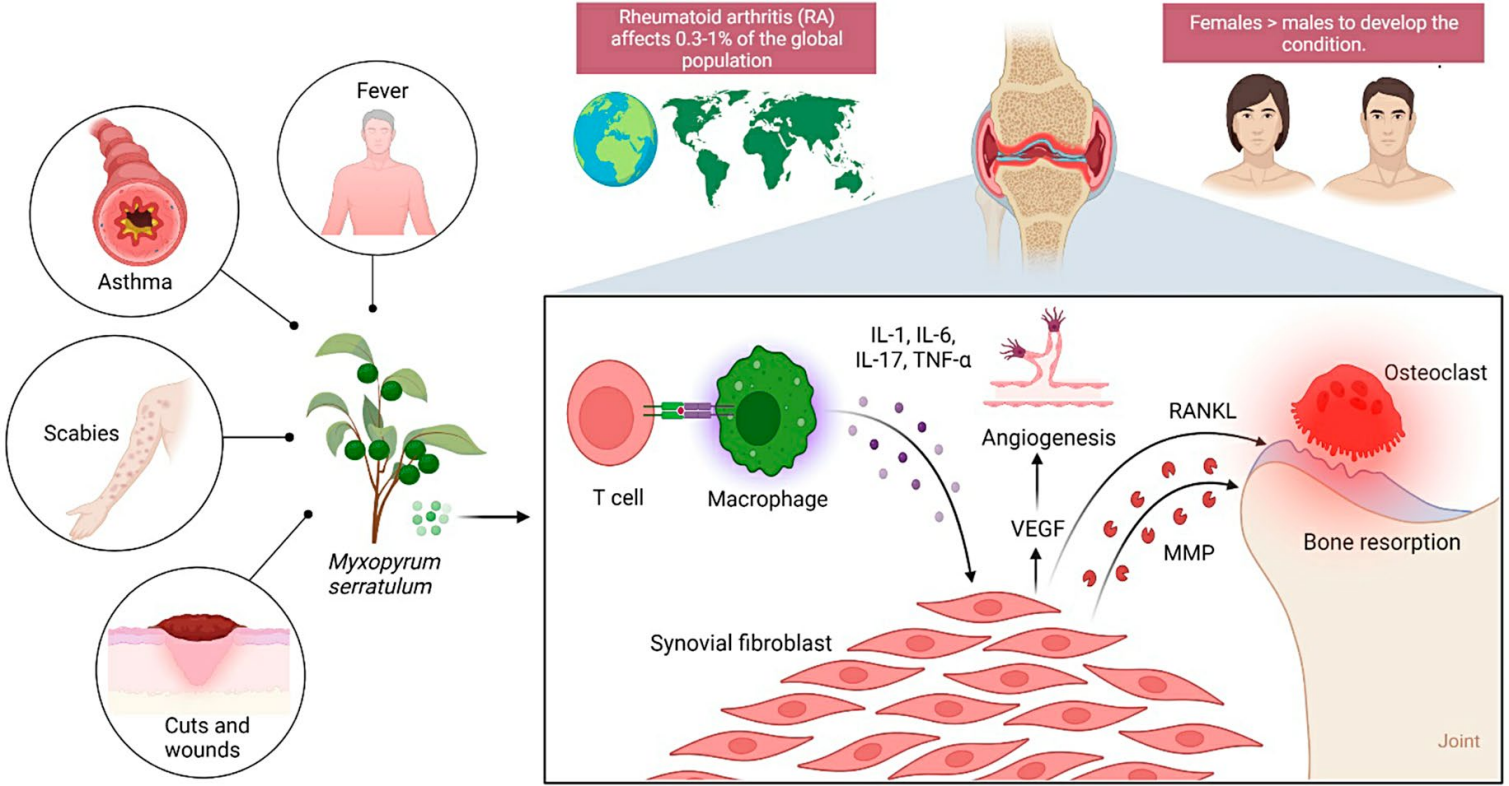
Mechanism of inflammation induced by pro-inflammatory cytokines in rheumatoid arthritis. *Abbreviations* Interleukins − 1, -6, -17, IL-1, -6, -17; Tumour necrosis factor-alpha, *TNF-α* Vascular endothelial growth factor, *VEGF* Receptor activator of nuclear factor kappa beta ligand, *RANKL* Matrix metalloproteinase, MMP

A systemic autoimmune disease known as rheumatoid arthritis (RA) is characterized by a chronic inflammatory process that causes joint damage, deformity, disability, and occasionally even death [1]. The rate of this illness ranges from 0.5 to 2%, and it is more common in women, smokers, and people with a family history of the condition [2]. RA primarily damages joint synovial membranes, which destroys the bone and cartilage. Globally, approximately 1% of adults in their 4-6th decades of life suffer from RA [3, 4]. Many genetic and environmental variables that influence the phenotype in various ways are linked to RA. Although the specific pathophysiology remains unknown, it is plausible that RA is impacted by the release of some free radicals such as superoxide and nitrous oxide radicals which are metabolic by-products of cellular metabolism [4]. Additionally, interleukin (IL) and tumour necrosis factor (TNF) production from T-cells may be stimulated by the release of such free radicals. The released proteins may in turn affect the production of growth factors, cytokines and adhesive molecules on immune cells, which may contribute to tissue damage and inflammation [5–7] (Fig. 1). It has been reported that approximately 1% of individuals worldwide have RA [3] and the disease is 2–3 times more common in women [8]. Cathepsin D is a lysosomal protease that plays a significant role in various physiological and pathological processes, including RA. In RA, cathepsin D has been implicated in several aspects of the disease pathogenesis such as joint destruction, inflammation and synovial hyperplasia, angiogenesis, autoimmunity and antigen presentation, and bone resorption [9].

Non-steroidal anti-inflammatory drugs (NSAIDs), glucocorticoids and disease-modifying antirheumatic medications (DMARDs) are only some pharmacological choices available against RA [10, 11]. Many RA patients continue to report symptoms including pain and fatigue following drug therapy [12]. Some medicines confer negative effects when administered over extended periods [13] indicating that there is an urgent need for the development of novel agents based on complementary and alternative medicine. Additionally, according to established research, medicinal plants and their constituents may be an effective adjuvant in reducing the symptoms of RA [14].

*Myxopyrum serratulum* A.W. Hill (*M. serratulum*) is belongs to the family of Oleaceae shown great interest since all plant parts including the root, stem and leaves have medicinal properties and are utilised in many traditional systems of medicine. The ethnomedicinal use include treatment of headaches, asthma, cough, fever, otitis, rheumatism and wounds. Traditionally, dried and powdered leaves of the *M. serratulum* are mixed with ghee as a remedy for asthma, cough and nerve complaints, apart from which they were also used for treating fever, headache and ear diseases. The plant’s berries were also reported for their anti-inflammatory activities [15–17].

According to Maruthamuthu et al. (2020), the methanolic extract of *M. serratulum* leaves contains the phytochemicals like gallic acid, protocatechuic acid, catechin, ellagic acid, rutin, p-coumaric acid, quercetin, naringenin and apigenin [15]. Rajalakshmi et al. (2016a) reported GC-MS analysis of ethanolic extract of *M. serratulum* leaves and reported 24 compounds which includes mainly 2-Propenoic acid, 3-(4-methoxy phenyl)-, 1-(2-Hydroxy-ethyl)-2-methyl-1 H-benzoimidazole-5-carboxylic acid methyl ester, n-Hexadecanoic acid, Isoquinoline, 1,2,3,4-tetrahydro-1-ally1-6,7-dimethoxy-3,3-dimethyl-, Phytol, 3,7,11,15-Tetramethyl-2-hexadecen-1-ol, Ethyl α-d-glucopyranoside, Pyrazolidine-3,5-dione, 4-phenyl-, Hexadecanoic acid, ethyl ester, 1 H-Indol-4-ol, 9,12-Octadecadienoic acid (z, z)-, 4-((1E)-3-Hydroxy-1-propenyl)-2-methoxyphenol, and oleic acid [18]. Further, Rajalakshmi et al. (2016b) reported GC-MS analysis of ethanolic extract of *M. serratulum* stem and reported 32 compounds which includes mainly 2-Propenoic acid, 3-(4-methoxyphenyl)-, α-Amyrin, 1-(2-Hydroxy-ethyl)-2-methyl-1 H-benzoimidazole-5-carboxylic acid methyl ester, n-Hexadecanoic acid, Pyrazolidine-3,5-dione and 4phenyl-, 5-hydroxymethylfurfural [19].

Preliminary research on *M. serratulum* stem and leaf extracts revealed that they had potent anti-inflammatory effects and great potential as a source of drugs for the treatment of inflammatory-related disorders [20].

Other than the above reported studies, no other phytoconstituents are known or documented in *M. serratulum* and the anti-inflammatory and anti-arthritic properties of *M. serratulum* have not yet been thoroughly researched. Therefore, the defatted ethanolic extract of *M. serratulum* (EEMS) have been investigated for its anti-inflammatory and anti-arthritic properties.

## Methods

### Collection, authentication and preparation of extract

Fresh plant was collected from Tirunelvelli, Tamilnadu, India and was authenticated by a botanist Prof. Jayaraman from the Plant Anatomical Research Centre, Tambaram, Chennai (Voucher specimen number: PARC/13/3604). The aerial parts were shade dried and coarsely powdered. About 2.5 kg of the powdered plant material was extracted successively by cold maceration method with different solvents (10 L each for 24–36 h) of increasing polarity (petroleum ether, chloroform, ethyl acetate and ethanol). After six days of cold maceration, all the extracts were concentrated under controlled pressure and temperature using rotary evaporator. The percentage yield for petroleum ether, chloroform, ethyl acetate and ethanol extracts were found to be 0.34, 0.95, 1.4, and 2.3%, respectively. All the extracts were stored in a desiccator until further use.

In the earlier studies, based on the GC-MS profile, it was reported that the large number of active constituents present in ethanolic extract of *M. serratulum* [18, 19]. Hence, in the present study, the defatted EEMS was selected to evaluate for its anti-inflammatory and anti-arthritic properties.

### Institutional Animal Ethical Committee approval

The study protocol was approved by the Institutional Animal Ethics Committee (IAEC/XXXXII/SRU/398/15) of Sri Ramachandra University, Chennai, India. The study was performed as per the guidelines of the Committee for the Purpose of Control and Supervision on Experiments on Animals (CPCSEA).

### Acute oral toxicity study

The healthy Wistar rats, 12–15 weeks old were divided into two groups (*n* = 3). EEMS (2000 mg/kg) was orally administered to groups I and II following gastric intubation. Observation for lethality and abnormal clinical symptoms (including behavioral abnormalities, movement, convulsions) were conducted on the first day up to 14 days. The body weight was measured at baseline and then every subsequent until the experimental period was over.

### Anti-inflammatory activity of EEMS

In order to eliminate the stress effect, the animals were acclimatized to standard husbandry conditions for one week prior to the experiments. Following acclimatization, the animals were randomized into five groups (Groups I –V) of six animals per group and were fasted overnight.

Group I – CMC (0.5%) + 0.1 mL of 1% carrageenan (Negative control).

Group II – Standard diclofenac (25 mg/kg, p.o.) + 0.1 mL of 1% carrageenan (Positive control).

Group III – EEMS (100 mg/kg, p.o.) + 0.1 mL of 1% carrageenan.

Group IV – EEMS (200 mg/kg, p.o.) + 0.1 mL of 1% carrageenan.

Group V – EEMS (400 mg/kg, p.o.) + 0.1 mL of 1% carrageenan.

All of the animal’s paw thicknesses were measured at “0 hour,” or right before to the carrageenan injection, and this value is considered as normal. Subsequently, 30–40 min after drug/vehicle treatment, a 1% λ-carrageenan (0.1 mL) was prepared in normal saline. The solution was injected into the plantar region of the right hind paw to induce edema [21]. Following carrageenan injection, the paw volume was measured at several different time intervals (0.5, 1, 2, 3 and 5 h) using a digital plethysmometer (Ugo Basile, Italy). Then, the mean value of three readings was taken. The estimated edema volume and the percentage of anti-inflammatory activity were calculated. Diclofenac (25 mg/kg) was used as a standard. The dose for the efficacy study was fixed based on the acute toxicity cut-off value of EEMS as well as the findings from the pilot study where 100, 200, 400 and 1000 mg/kg doses were tested. Since the dose above 400 mg/kg yielded a ceiling effect, a fixed dose (100, 200 and 400 mg/kg) were selected and compared to the standard diclofenac (25 mg/kg).

### Anti-arthritic activity of EEMS

The inflammatory agent known as Complete Freund’s adjuvant (CFA) causes the production of antigenic molecules in both joints and tissue. CFA-induced paw inflammation resembles rheumatoid arthritis in humans. There were six animals in each of the six groups.

#### Group I (Normal control)

Animals in this group were administered a solution of 0.5% w/v carboxymethyl cellulose (CMC).

#### Group II (Diseased control)

This group received 0.1 mL of Complete Freund’s Adjuvant (CFA) emulsions at a concentration of 5 mg/mL, combined with 0.5% w/v of carboxymethyl cellulose CMC.

#### Group III (Positive control)

To serve as a positive control, animals in this group were treated with 0.1 mL of Complete Freund’s Adjuvant (CFA) emulsions (5 mg/mL) in combination with 0.1 mg/kg of methotrexate administered intraperitoneally.

#### Group IV (EEMS 100 mg/kg)

Animals in this group received 0.1 mL of Complete Freund’s Adjuvant (CFA) emulsions (5 mg/mL) followed by an oral dose of EEMS at 100 mg/kg.

#### Group V (EEMS 200 mg/kg)

Similar to Group IV, this group was administered 0.1 mL of Complete Freund’s Adjuvant (CFA) emulsions (5 mg/mL) but received a medium dose of EEMS at 200 mg/kg orally.

#### Group VI (EEMS 400 mg/kg)

This group received 0.1 mL of Complete Freund’s Adjuvant (CFA) emulsions (5 mg/mL) along with the highest oral dose of EEMS at 400 mg/kg.

Adjuvant arthritis was produced by injecting 0.1 mL of CFA emulsions (5 mg/mL) into the right hind paw’s sub-plantar region [22]. Both drugs and vehicles were administered for 21 consecutive days. The paw volume was assessed at various time points including 0, 3, 6, 9, 12, 15 and 21st days following CFA injection [23].

The body weight was measured every week for 21 days. On the last day, the animals were anesthetized using isoflurane. Blood was collected for biochemical analysis via a retro-orbital puncture and was stored. The animals were euthanized and the liver was stored for antioxidant studies. The paw tissue was utilized for the histopathology, cathepsin-D content and gene expression studies. Immediately following sacrifice, the organs like the liver, kidney, brain, spleen and testes were excised, rinsed with saline, blotted dry using a filter paper and were weighed. A 10% w/v liver tissue homogenates were prepared with an appropriate buffer using a tissue homogenizer. The homogenate was centrifuged at 1000 rpm for 10 min at 4 °C in a cooling centrifuge and subsequently used for various antioxidant estimations. Tissue (1 g) was scrapped from the dissected paws and homogenized in a 10 mL of potassium chloride (0.1 M) to prepare a tissue homogenate, which was used for experiments.

#### Biochemical parameters

Biochemical parameters such as serum glutamate oxaloacetate transaminase (SGOT), serum glutamate pyruvate transaminase (SGPT), serum creatinine, C-reactive protein (CRP), serum urea and total protein were assayed using commercial diagnostic kits (Accurex Diagnostics, India).

#### Estimation of tissue lipid peroxide (LPO) level

The tissue lipid peroxide (LPO) level was determined as TBA-reactive substances according to the method of Ohkawa et al., 1979 [24]. The method involved heating the sample (0.2 mL) with saline (0.8 mL), butylated hydroxytoluene (0.5 mL) and thiobarbituric acid reagent (35 mL) for 15 h in a boiling water bath. After cooling, the solution was centrifuged at 3500 rpm for 10 min and the precipitate was removed. The absorbance of the supernatant was measured at 532 nm using a spectrophotometer. The values were expressed as nanomoles/gm tissue.

#### Estimation of superoxide dismutase

In order to measure superoxide dismutase (SOD) levels, the sample (0.05 mL) was mixed to 0.3 mL of sodium pyrophosphate buffer (0.025 M, pH 8.3), 0.025 mL of phenazonium methosulphate (186 µM) and 0.07 mL of nitroblue tetrazolium chloride (300 µM in a buffer of pH 8.3). The reaction was started by adding 0.075 mL of a reduced nicotinamide adenine dinucleotide (780 µM in a buffer of PH 8.3). The reaction was halted by adding 0.25 mL glacial acetic acid following incubation at 30 °C for 90 s. Next, n-butanol (2 mL) was added and the reaction mixture was vigorously agitated. The mixture was centrifuged after being left to stand for 10 min. The blank was a pure n-butanol (1.5 mL) while a UV spectrophotometer was used to measure the chromogen’s colour intensity at 560 nm [25].

$${\rm{Enzyme}}\,{\rm{activity}}\,\left( {1{\rm{ unit}}} \right)\, = \,50\% \,{\rm{inhibition}}/{\rm{min}}$$

#### Estimation of glutathione peroxidase (GPx)

To determine glutathione peroxidase activity (GPx), a slightly modified method of Rotruck et al. (1973) was used [26]. Briefly, 200 µl of tris-hydrochloric acid buffer (0.4 M), 200 µl of potassium EDTA (0.4 mM), 100 µl of sodium azide and 200 µl of sample were well-combined. Thereafter, 200 µl of reduced glutathione solution (2 mM) and hydrogen peroxide (0.1 mL) were added. The mixture was left to sit for 10 min at 37 °C. Then, 0.5 mL of 10% trichloroacetic acid (TCA) was added to stop the reaction. The solution was centrifuged (10 min at 1500 rpm) to separate the precipitate. A spectrophotometer was used to measure the colour intensity at 412 nm that resulted after adding 1.0 mL of di-thio nitrobenzoic acid (DTNB) and 0.5 mL of saline to 0.2 mL of the supernatant.

#### Estimation of reduced glutathione (GSH)

Reduced glutathione was measured by adding 0.25 mL of the sample to an equivalent volume of ice-cold 5% TCA. This was followed by a centrifugation step for 10 min at 3500 rpm, done to separate the precipitate. Subsequently, a 0.2 M phosphate buffer, pH 8.0 and 0.5 mL of DTNB (0.6 mM in 0.2 M phosphate buffer, pH 8.0) were added. The solution was thoroughly mixed following the addition of the supernatant (1 mL) and the absorbance measured at 412 nm [27].

#### Estimation of Cathepsin-D

A buffered substrate and 0.5 mL of the sample were combined, were thoroughly mixed and incubated (45 °C for two hours). The process was stopped with the addition of 10% TCA (750 µl). The tubes were incubated for an hour at room temperature. The reaction mixture was centrifuged at 3500 rpm for 15 min. Then, 1.5 mL of Folin’s phenol reagent was added (1:2), followed by 2 mL of supernatant, 3 mL of sodium hydroxide (0.5 N) and 2 mL of supernatant. The absorbance was measured at 620 nm against a blank (0.5% CMC). Tyrosine was plotted on a standard graph and the amount of liberated tyrosine was calculated [28].

### Gene expression study of inflammatory marker-RT PCR

A reverse transcriptase-polymerase chain reaction (RT-PCR) was performed as described by Bang–Tian Chen et al [29]. Briefly, TRIZOL Reagent was used to quickly extract the total RNA from scraped tissues of the dissected paw (Sigma, USA). The tubes were filled with homogenized tissues, then incubated for 10 min, followed by a 5 min centrifugation at 1000 rpm. The supernatant was mixed with chloroform (200 µl), followed by incubation at room temperature for 5 min and a final centrifugation (20 min at 12000 rpm). Following a 10-minute incubation period, isopropyl alcohol (500 µl) was added to the supernatant to precipitate the total RNA. The mixture was centrifuged at 12,000 rpm for 15 min. The supernatant was carefully removed and the pellet rinsed three times in 75% ethanol, centrifuged at 12,000 rpm for 15 min and then allowed to air dry. Prior to use, the pellet was re-suspended in RNase-free water (20 µl) and kept at -80^o^C. The extracted RNA will be used for reverse transcription and polymerization using a Gradient’s PCR master cycler to produce cDNA. The bands created during the agarose gel (2%) electrophoresis were used to assess the gene expression. Below are the details of the primer sequence employed in this study.

Inducible Nitric Oxide Synthase (iNOS): The primer sequence used for detecting iNOS expression included a sense strand, starting with the sequence “5’-AAT GGC AAC ATC AGG TCG GCC ATC ACT-3’.” This sequence was designed to perfectly match the target RNA, enabling precise amplification of the iNOS gene. The corresponding anti-sense strand, “5’-GCT GTG TGT CAC AGA AGT CTC GAA CTC-3’,” complements the sense strand, ensuring accurate detection and quantification of the gene’s expression levels.

Interleukin-1 Beta (IL-1β): To assess the levels of IL-1β, a cytokine crucial in mediating inflammatory responses, the study employed a sense primer with the sequence “5’-TGC AGA GTT CCC CAA CTG GTA CAT C-3’.” This sequence aligns with the mRNA of IL-1β, facilitating its detection. The anti-sense primer, “5’-GTG CTG CCT AAT GTC CCC TTG AAT C-3’,” pairs with the complementary strand, providing a robust mechanism for evaluating the gene’s expression in inflamed tissues.

Tumour Necrosis Factor Alpha (TNF-α): TNF-α, a potent pro-inflammatory cytokine, was targeted using a sense primer with the sequence “5’-ATG AGC ACA GAA AGC ATG ATC-3’.” This primer sequence was meticulously chosen to bind specifically to TNF-α mRNA, ensuring reliable amplification. The anti-sense primer, “5’-TAC AGG CTT GTC ACT CGA ATT-3’,” complements the sense strand, enabling precise measurement of TNF-α levels in the experimental samples.

Glyceraldehyde 3-Phosphate Dehydrogenase (GAPDH): GAPDH was employed as a housekeeping gene to normalize the expression of the target genes. The sense primer sequence “5’-TCA TGA AGT GTG ACG TTG ACA TCC GT-3’” was used to detect GAPDH mRNA, ensuring consistent and reliable baseline readings. The corresponding anti-sense primer, “5’-CCT AGA AGC ATT TGC GGT GCA CGA TG-3’,” provided a stable reference point, ensuring that the quantification of other genes was accurate and comparable across samples.

### Histopathological studies

All of the animals were sacrificed using isoflurane after 21 days. The animals had their right hind paws severed close to the tibiotarsal join. The tissue was fixed in a 10% neutral buffered formalin for a whole day. With the use of hydrochloric acid and EDTA solution (0.1 M), the paws were decalcified. The ankle joints were divided in half in the longitudinal plane upon completion of the decalcification procedure. The distal tibia indicated both cortices and an abundance of medullary space and the articular surfaces of the opposing bones could be seen when the ankle joints were prepared for paraffin embedment and sectioned. Paw tissue representative samples from each group were gathered, dried in a succession of progressively stronger alcohols and were prepared for paraffin embedment. For a general histological assessment of arthritis, tissue slices were prepared and stained with hematoxylin and eosin [30, 31].

### Statistical analysis

The data were reported as mean ± standard error mean (SEM) for all the activities. To compare the means of all treatment groups, a one-way ANOVA was employed followed by a multiple comparison test (Tukey-Kramer). Confidence levels of 99% (*p* < 0.01) and 95% (*p* < 0.05) were considered as statistically significant. A graphPad Prism (Version 5.0, Boston MA, USA) was used for all analysis.

## Results

### Acute oral toxicity of EEMS

None of the experimental animals experienced treatment-related mortality, unusual clinical symptoms, notable changes in body weight, or obvious pathological alterations. No mortality was noted upon EMS administration of up to 2000 mg/kg. An increase in the body weight occurred from day 0–14) confirming that EEMS does not cause any apparent acute toxicity. The LD_50_ was greater than 2000 mg/kg which makes it in the “category 5” or “unclassified” based on the Globally Harmonised System (GHS).

### Anti-inflammatory activity of EEMS

**Fig. 2:**
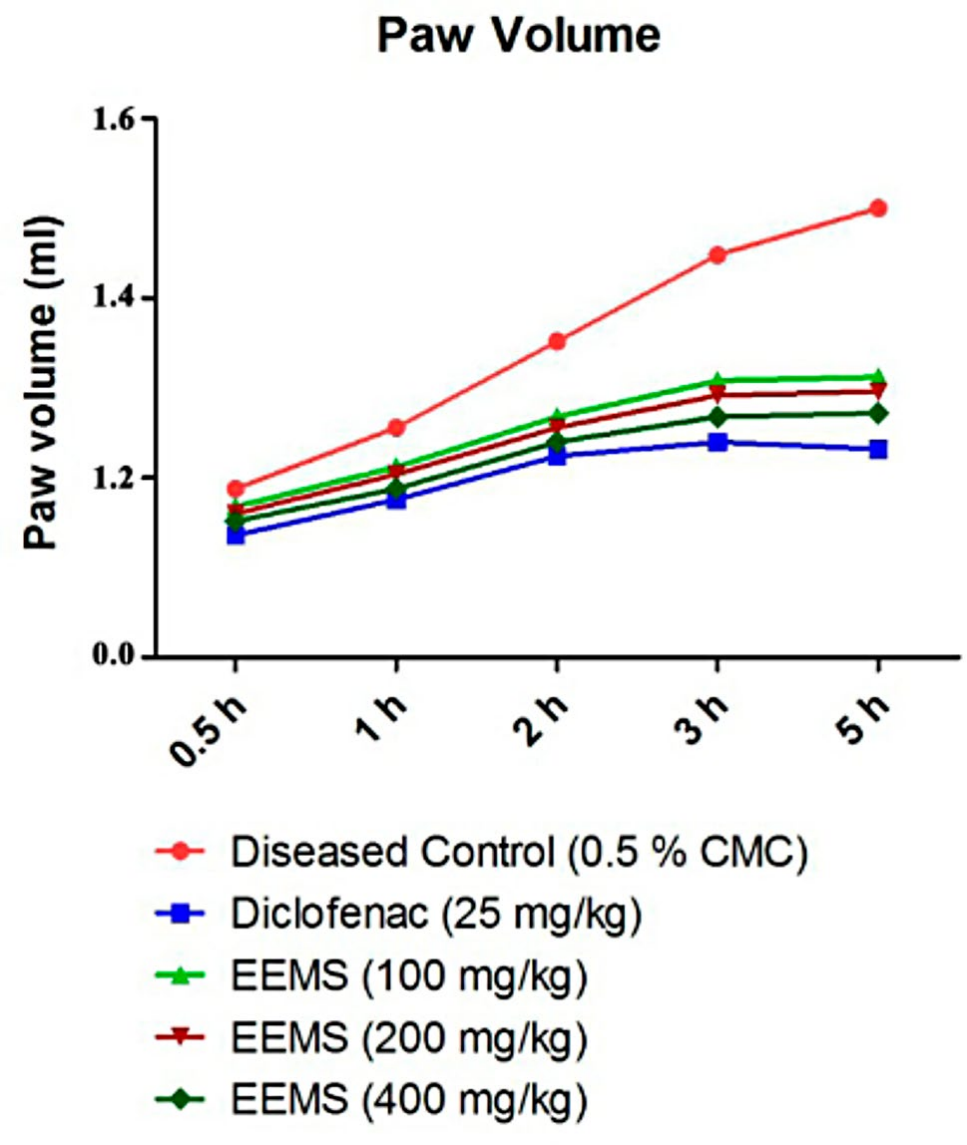
Effect of EEMS on the paw volume of carrageenan-induced rats

The anti-inflammatory activity of EEMS was investigated at three different doses (100, 200 and 400 mg/kg, p.o.) in Sprague-Dawley rats by carrageenan-induced paw edema. The reduction in the paw diameter after administration of the different treatments at the various time intervals was plotted (Fig. 2). In the diseased group, the paw volume was increased till the 5th hour. However, similar as the control group, the EEMS-treated group (100, 200 and 400 mg/kg) showed an increase in the paw volume till the 2nd hour, followed by a reduction in the 3rd and 5th hour.

### Anti-arthritic activity of EEMS

#### Estimation of body weight

Body weight is a non-specific indicator of the animal’s general well-being where its reduction indicates an effect on the general health. In our study, the body weight was measured at several time intervals (days 0, 7, 14 and 21) (Table 1). CFA injection produced a significant but persistent weight loss. In the EEMS-treated group, no significant change was seen in the body weight until the second week (14 days). In comparison, animals in groups 5 and 6 which received EEMS (200 and 400 mg/kg) respectively, experienced a significant increase in the body weight in the third week as compared with the standard drug-treated group. On the other hand, a mild reduction in the body weight was observed in the arthritic-control, methotrexate and restoration by EEMS-treated groups.

**Table 1:** Effect of EEMS on the body weight of CFA-induced arthritic rats

Treatment	Body weight (g)			
	Week 0	Week 1	Week 2	Week 3
Normal control (0.5% CMC)	162.00 ± 3.45	166.83 ± 3.83	174.83 ± 4.00	182.00 ± 4.10
Diseased control (CFA)	153.00 ± 2.77	164.50 ± 2.93	157.00 ± 3.61^a*^	149.67 ± 3.87^a*^
Methotrexate (0.1 mg/kg, i.p.)	160.83 ± 5.53	172.00 ± 3.15	164.00 ± 3.44	155.17 ± 3.74
EEMS (100 mg/kg, p.o.)	153.00 ± 3.50	164.67 ± 3.37	160.17 ± 4.06	154.50 ± 3.71
EEMS (200 mg/kg, p.o.)	155.83 ± 2.81	168.00 ± 2.14	168.17 ± 1.58	172.17 ± 1.70^b*^
EEMS (400 mg/kg, p.o.)	155.40 ± 7.30	166.00 ± 6.23	165.83 ± 8.62	179.17 ± 8.22^b**^

Values were expressed as mean ± Standard Error Mean (SEM); *n* = 6 animals; A One-way ANOVA followed by Tukey-Kramer multiple comparison test was used to compare the means of all the treatment group. a: Comparisons between normal control with diseased control. b: Comparisons between diseased control with standard methotrexate (0.1 mg/kg, i.p.) and EEMS (100, 200, 400 mg/kg) treated groups. p-value: [* represents *p* < 0.05 and ** represents *p* < 0.01]

### Estimation of paw volume

**Fig. 3:**
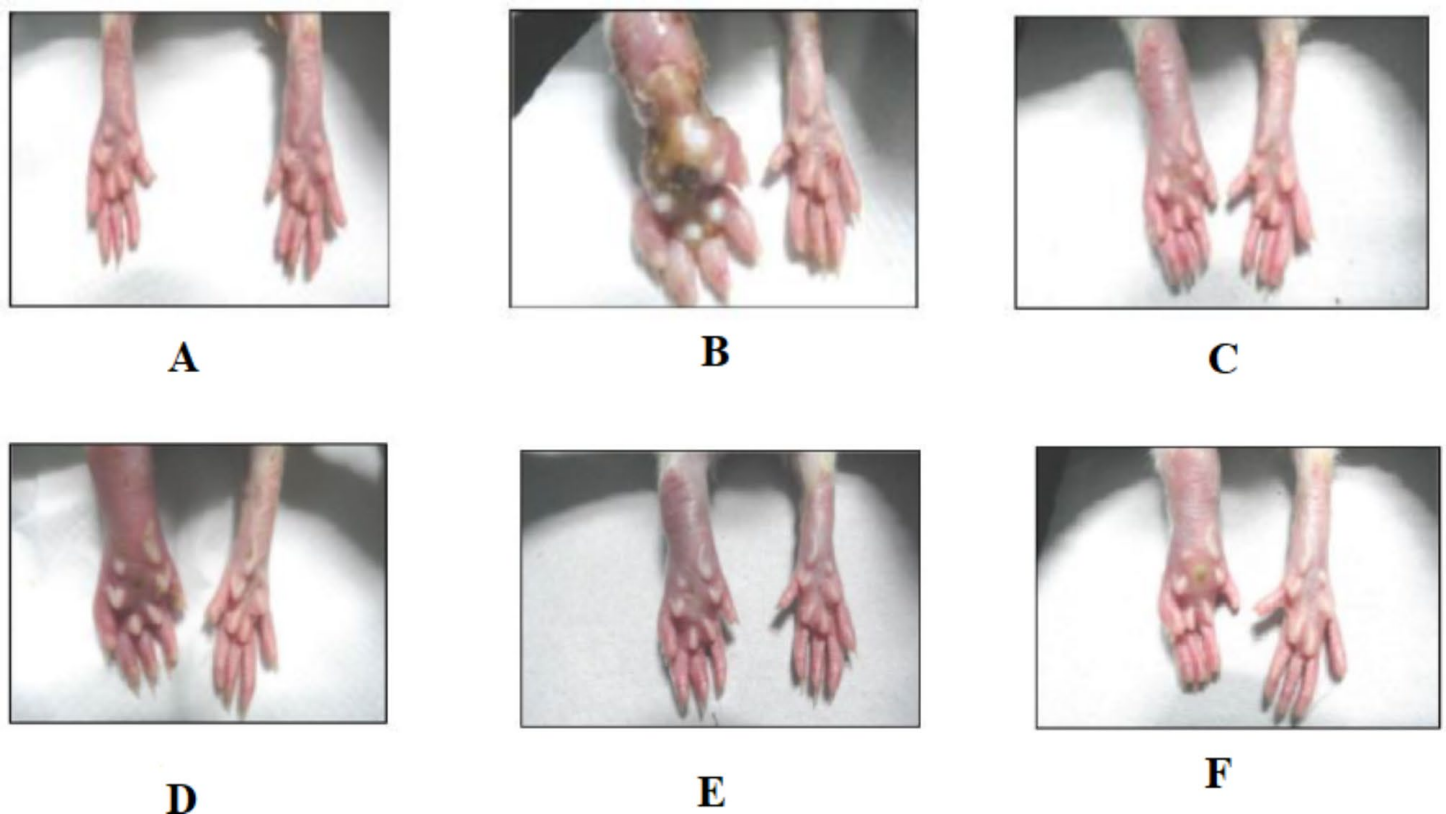
Photographs of the rats hind paw induced by CFA and the effect of EEMS treatments. **(A)** normal control **(B)** diseased control **(C)** diclofenac (25 mg/kg) **(D)** EEMS (100 mg/kg) **(E)** EEMS (200 mg/kg) and **(F)** EEMS (400 mg/kg)

**Fig. 4:**
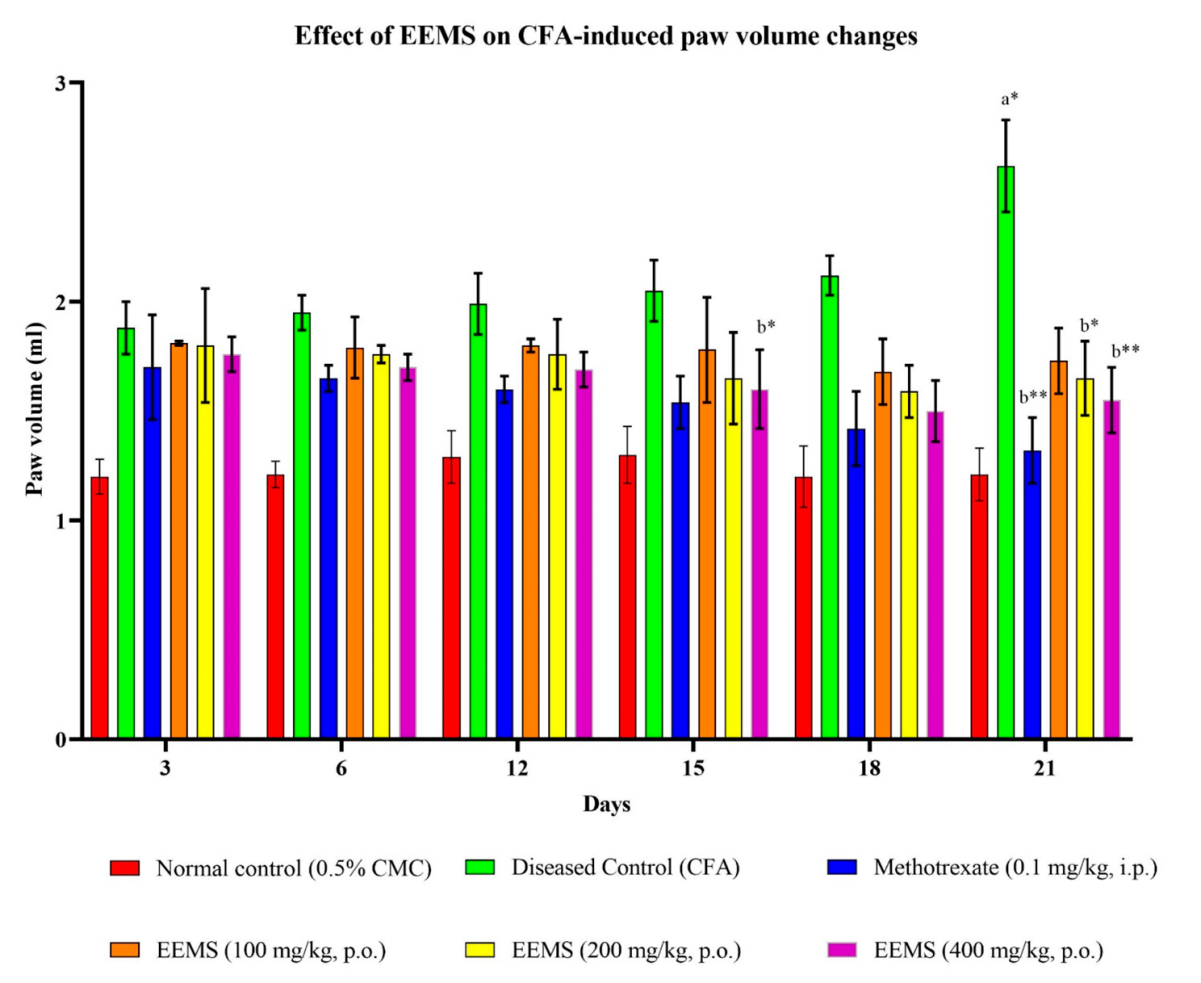
Effect of EEMS on CFA-induced paw volume changes. Values were expressed as mean ± Standard Error Mean (SEM); *n* = 6 animals; A one-way ANOVA followed by Tukey-Kramer multiple comparison test was used to compare the means of all treatment groups. a: Comparisons between normal control and diseased control. b: Comparisons between diseased control with standard methotrexate (0.1 mg/kg, i.p.) and EEMS (100, 200, 400 mg/kg)-treated groups. p-value: [*represents *p* < 0.05, **represents *p* < 0.01]

The arthritic control group’s paw edema was significantly increased following CFA injection (Fig. 3). Rats administered with CFA demonstrated soft tissue edema around the ankle joints as the arthritic disease progressed. A progressive increase in the volume of the injected paw further illustrates chronic inflammation in the CFA model. Paw enlargement is an index to measure of anti-arthritic activity. The paw volume was measured on 3rd, 6th, 9th, 12th, 15th, 18th and 21st days (Fig. 4). Based on our study, there was amelioration in the paw volume due to EEMS administration in addition to the standard methotrexate. Paw edema was dramatically reduced following methotrexate treatment (59.60%, *p* < 0.01). Similarly, its development was significantly reduced after EEMS treatment. On the other hand, the paw volume of the arthritis control group significantly increased, demonstrating the onset of arthritis. Higher EEMS dose (400 mg/kg) tend to cause a greater paw volume reduction (49.01%) as compared to that for 200 mg/kg (45.05%) and 100 mg/kg (42.05%) indicating that the effect is dose-dependent.

#### Biochemical estimations

Table 2 shows biochemical changes including SGOT, SGPT, total protein, urea, creatinine and CRP levels. Based on our study, the levels of SGOT and SGPT in the arthritic control group were higher than that for the standard control indicating some liver damage. The EEMS (400 mg/kg)-treated group displayed the most significant reduction in the SGOT and SGPT values, whereas methotrexate therapy resulted in an increase in both values. Additionally, there were higher blood urea and serum creatinine levels in the arthritic control group suggesting some kidney impairment resulting from kidney injury and/or impaired glomerular function. CRP is frequently used to indicate systemic inflammation. CRP levels in the arthritic control group were significantly increased. On the other hand, CRP level was significantly decreased in both the extract-treated and standard drug control groups. Again, the highest EEMS dose (400 mg/kg) significantly (*p* < 0.01) lowered CRP levels.

**Table 2:** Effect of EEMS on biochemical parameters in CFA-induced arthritic rats

Treatment	Biochemical Parameters					
	SGOT (U/L)	SGPT (U/L)	Creatinine (mg/dl)	Urea (mg/dl)	C-Reactive Protein (mg/dl)	Total Proteins (g/dl)
Normal control (0.5% CMC)	88.97 ± 14.36	33.25 ± 0.65	0.68 ± 0.04	35.60 ± 2.89	0.31 ± 0.01	5.78 ± 0.25
Diseased Control (CFA)	91.62 ± 14.30	31.25 ± 5.67	0.70 ± 0.07	36.34 ± 2.70	1.39 ± 0.23^a*^	6.12 ± 0.30
Methotrexate (0.1 mg/kg, i.p.)	114.34 ± 18.52	49.31 ± 9.28	1.03 ± 0.06	45.88 ± 2.72	0.34 ± 0.02^b**^	5.95 ± 0.19
EEMS (100 mg/kg, p.o.)	98.24 ± 14.66	31.13 ± 6.9	0.69 ± 0.04	39.98 ± 2.89	0.33 ± 0.01	6.29 ± 0.33
EEMS (200 mg/kg, p.o.)	102.14 ± 3.27	38.31 ± 8.32	0.63 ± 0.00	39.27 ± 3.63	0.36 ± 0.01^b*^	6.45 ± 0.29
EEMS (400 mg/kg, p.o.)	89.29 ± 12.99	34.86 ± 3.04	0.68 ± 0.41	34.62 ± 2.26	0.34 ± 0.02^b**^	6.37 ± 0.22

Values were expressed as mean ± Standard Error Mean (SEM); *n* = 6 animals; A one-way ANOVA followed by Tukey-Kramer multiple comparison test was used to compare the means of all treatment groups. a: Comparisons between normal control and diseased control. b: Comparisons between diseased control with standard methotrexate (0.1 mg/kg, i.p.) and EEMS (100, 200, 400 mg/kg) treated groups. p-value: [* represents *p* < 0.05 and ** represents *p* < 0.01]

### Effect on liver antioxidant parameters

#### SOD

**Fig. 5:**
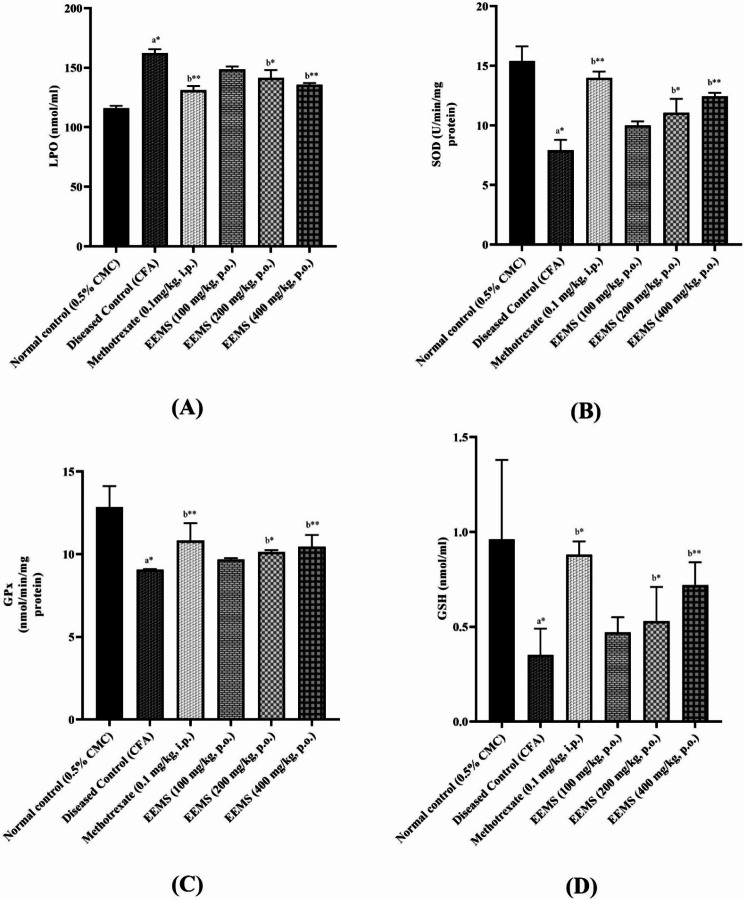
Effect of EEMS on Liver Antioxidant Levels in Arthritic Rats. **(A)** Lipid Peroxidation (LPO) Levels: This bar graph illustrates the levels of lipid peroxidation (measured in nmol/ml) across different treatment groups. The disease control group shows significantly elevated LPO levels compared to the normal control, indicating increased oxidative stress. Treatment with Methotrexate and various doses of Ethanolic Extract of [Substance] (EEMS) significantly reduced LPO levels, suggesting its protective effect against oxidative damage. *p < 0.05, **p < 0.01. **(B)** Superoxide Dismutase (SOD) Activity: The graph displays SOD activity (measured in EU/min/mg protein), reflecting the antioxidant defense mechanism against oxidative stress. The disease control group exhibits reduced SOD activity, while treatment with Methotrexate and EEMS showed a significant restoration of SOD activity. This suggests that EEMS helps in enhancing antioxidant defenses. *p < 0.05, **p  < 0.01. **(C)** Catalase (CAT) Activity: This bar graph represents the catalase activity (measured in μmol of H2O2/min/mg protein). The disease control group shows a significant reduction in CAT activity, indicating impaired antioxidant defense. Treatment with Methotrexate and EEMS demonstrated a dose-dependent increase in CAT activity, signifying the potential of EEMS to restore catalase function. *p < 0.05, **p < 0.01. **(D)** Glutathione (GSH) Levels: This graph highlights GSH levels (measured in nmol/ml), a key antioxidant molecule that mitigates oxidative stress. The disease control group shows decreased GSH levels, while treatment with Methotrexate and EEMS at various doses significantly elevated GSH levels, suggesting that EEMS helps replenish this critical antioxidant. *p < 0.05, **p < 0.01. Statistical Analysis: Data are expressed as mean ± standard error of the mean (SEM) with *n* = 6 animals per group. A one-way ANOVA followed by Tukey-Kramer multiple comparison test was used to compare the means of all treatment groups. Comparisons were made between the normal control and diseased control group (denoted as ‘a’), and between the diseased control group and the groups treated with standard Methotrexate (0.1 mg/kg, i.p.) and EEMS (100, 200, and 400 mg/kg, p.o.) (denoted as ‘b’). Statistical significance is represented by *p < 0.05 and **p < 0.01

A significant (*p* < 0.01) reduction in SOD level was observed in the arthritic control rats when compared to the normal rats. Treatment with EEMS significantly and dose-dependently improved SOD levels when compared to positive control. Again, the highest dose of EEMS (400 mg/kg) yielded a significant improvement in SOD level comparable to that of the standard arthritic drug (Fig. 5).

#### LPO

A significant (*p* < 0.01) elevation in LPO level was observed in the positive control group when compared to the normal rats. Rats which received EEMS showed a significant (*p* < 0.01) reduction in LPO levels when compared to the positive control. The effect seen was comparable to that of the standard drug methotrexate (Fig. 5).

#### Reduced glutathione (GSH)

A reduction in the reduced glutathione (GSH) level was observed in the positive control compared to normal control rats. On the other hand, a significant (*p* < 0.01) elevation in GSH level was observed in the EEMS-treated group. Overall, treatment with EEMS restored the damage caused due to arthritic induction (Fig. 5).

#### Glutathione peroxidase (GPx)

A significant (*p* < 0.01) reduction in the GPx level was observed in the positive control when compared to the normal rats. Overall, EEMS treatment reduced GPx levels. However, this time, the effect was not dose-dependent since only EEMS (200 mg/kg) yielded a significant (*p* < 0.01) elevated GPX levels when compared to positive control (Fig. 5).

#### Effect of EEMS on Cathepsin-D

**Fig. 6:**
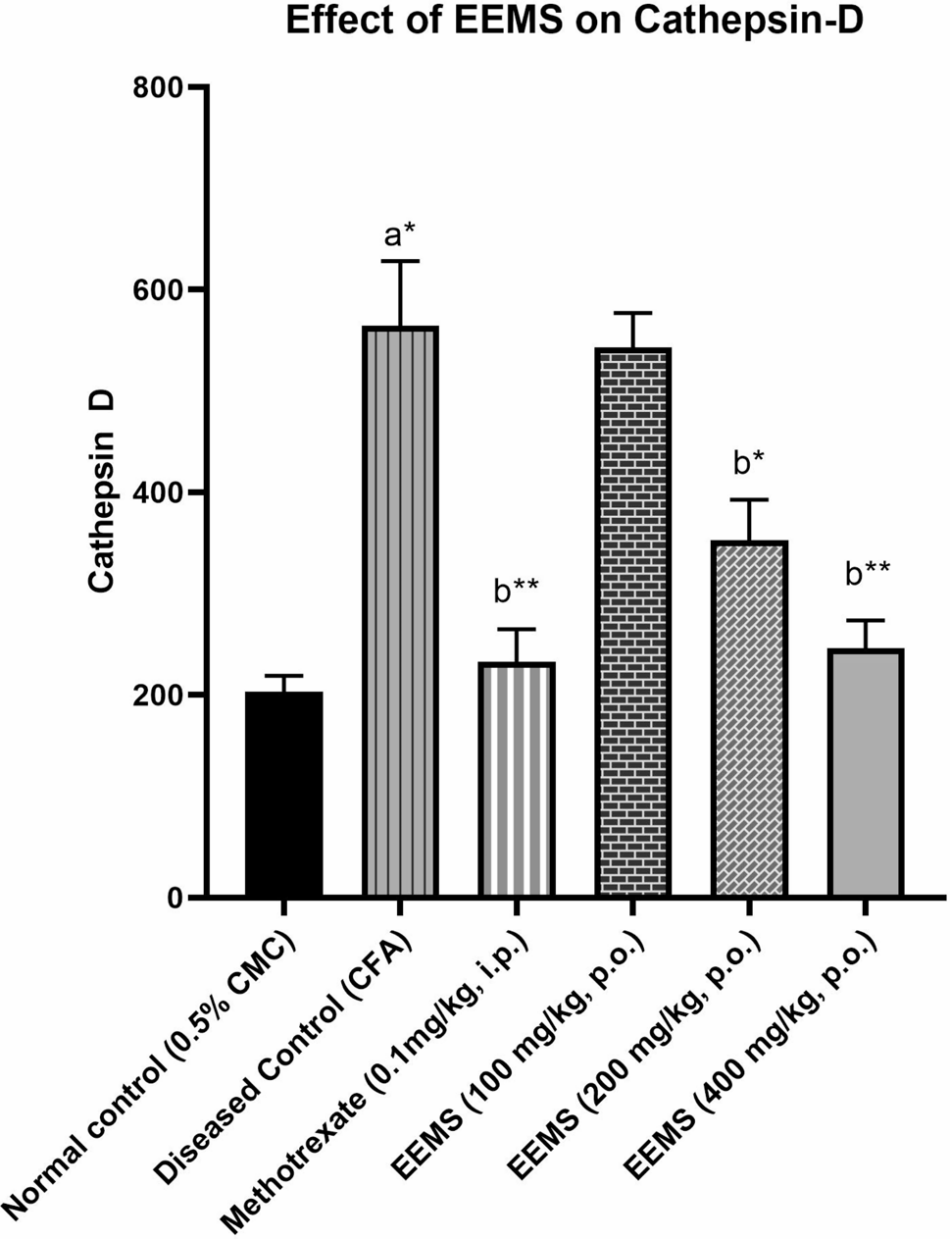
Effect of EEMS on Cathepsin-D. Values were expressed as mean ± Standard Error Mean (SEM); *n* = 6 animals; A one-way ANOVA followed by a Tukey-kramer multiple comparison test was used to compare the means of all treatment groups. a: Comparisons were made between normal control and diseased control. b: Comparisons between diseased control with standard methotrexate (0.1 mg/kg, i.p.) and EEMS (100, 200, 400 mg/kg) treated groups. p-value: [* represents *p* < 0.05, ** represents *p* < 0.01]

A significant (*p* < 0.01) elevation in cathepsin-D level was observed in the arthritic rats when compared to the normal rats. EEMS (400 mg/kg) caused a significant reduction in cathepsin-D level when compared to positive control. Treatment with EEMS significantly inhibited the accumulation of neutrophils at the inflammation site as evidenced by the decreased levels of cathepsin-D when compared with vehicle-treated rats (Fig. 6).

### Effect of EEMS on inflammatory markers in arthritic rats

**Fig. 7:**
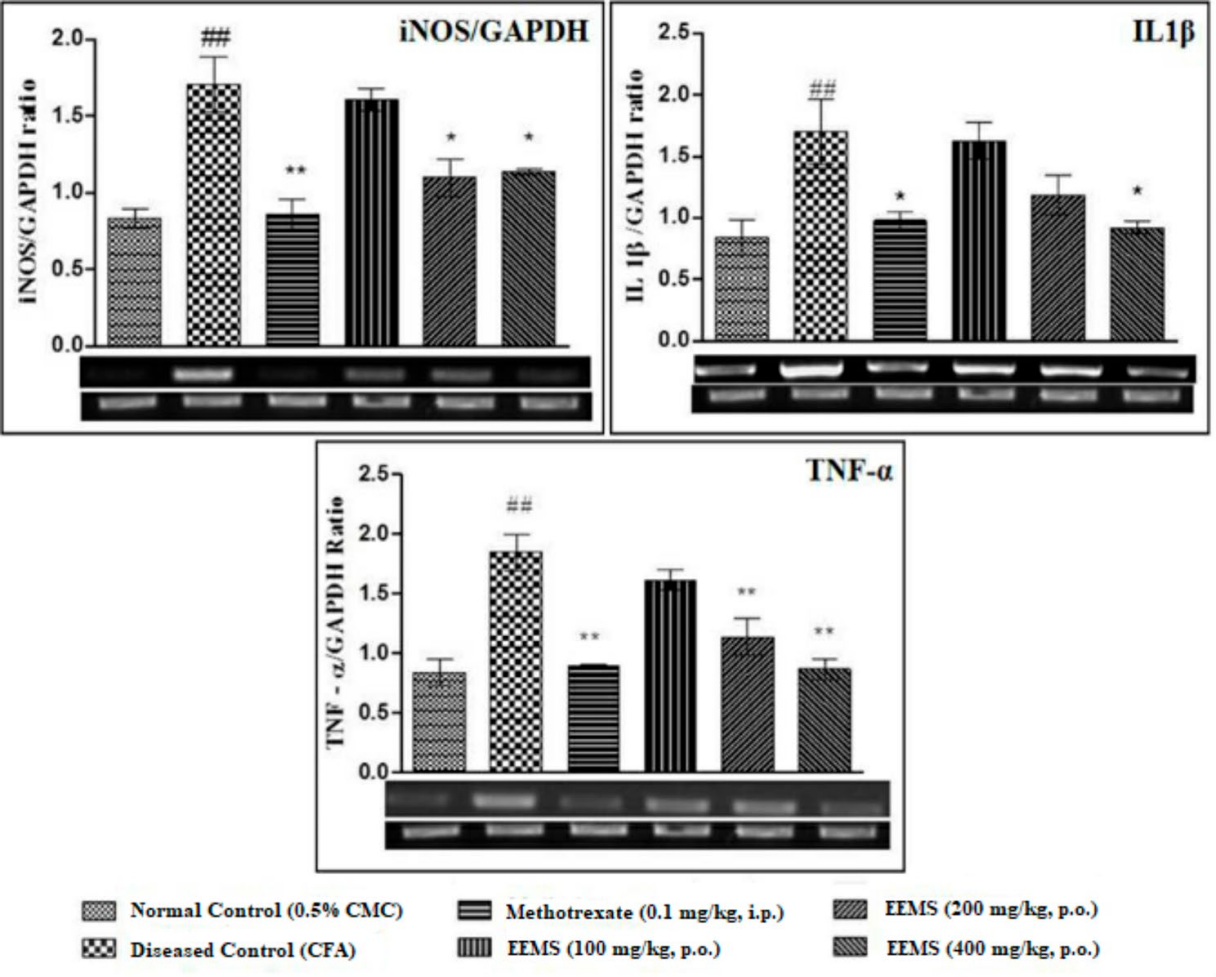
Effect of EEMS on inflammatory markers in arthritic rats. Values were expressed as mean ± SEM, *n* = 6 rats per group. ## represents *p* < 0.01 (## Comparisons were made between normal control and diseased control group); *represents *p* < 0.05, ** represents *p* < 0.01 (*Comparisons between diseased control with methotrexate (0.1 mg/kg) and EEMS (100, 200, 400 mg/kg groups). Abbreviation: iNOS: inducible nitric oxide synthase; GADPH: glyceraldehyde 3-phosphate dehydrogenase; IL1β- Interleukin-1 beta and TNF-α: tumor necrosis factor alpha

CFA administration produced a significant (*p* < 0.01) increase in the expression of iNOS/GADPH ratio, IL1β and TNF-α levels in the paw tissue homogenate of arthritic rats when compared to vehicle-treated rats. Oral administration of EEMS (200 and 400 mg/kg) equally reduced the iNOS/GADPH ratio levels in tissue homogenate when compared to the arthritic rats. At the same time, oral administration of 400 mg/kg EEMS yielded a significant reduction in IL1-β levels in the tissue homogenate compared to arthritic rats. In addition, oral administration of EEMS (200 and 400 mg/kg) significantly reduced TNF-α levels in the tissue homogenate in a dose-dependent manner when compared to arthritic rats (Fig. 7).

### Histopathology

**Fig. 8:**
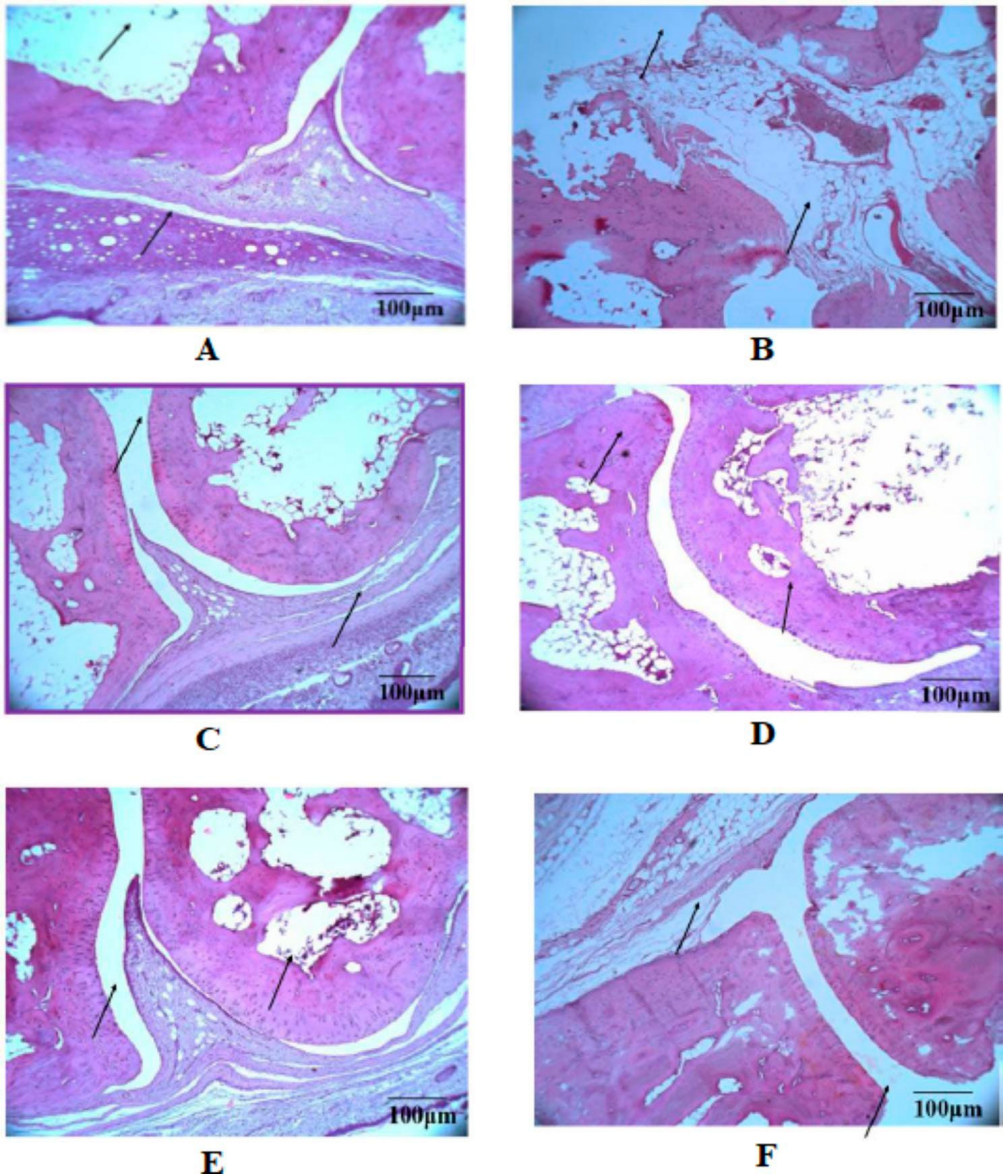
Effect of EEMS on inflammatory markers in arthritic rats. **(A)** Normal control: Bone and cartilage between the joints appeared normal. No pannus formation and no inflammatory cells infiltration seen. Synoviocytes surrounding the cartilage appeared normal. The fibrous joint capsule appeared normal. **(B)** Diseased control: Bone destruction. Severe infiltration of the synovium with inflammatory cells. Extensive proliferation of fibrovascular tissue. Erosion of joint cartilage and bone). **(C)** Methotrexate (0.1 mg/kg): Bone and cartilage surrounding the joints appeared normal. Synovium, fibrous joint capsule and tendon sheath appeared normal. No inflammatory cell infiltration in the synovium). **(D)** EEMS (100 mg/kg): Moderate bone destruction and mild cartilage erosion noticed. **(E)** EEMS (200 mg/kg): Degeneration of articular Cartilage and synovial membrane. The bone structure appeared normal, with no erosion. **(F)** EEMS (400 mg/kg): The bone structure appeared normal, with no destruction of bone. Articular cartilage, fibrous joint capsule and tendon sheath become regenerated in a proper manner. No inflammatory cells infiltration in the synovium

Paw tissues of rats were stained using hematoxylin and eosin for histopathological changes. Figure 8 represents the changes in paw tissue with treatments of EEMS. Administration of the highest EEMS dose (400 mg/kg) yielded the most significant effect on the disease. The bone structure of the animals which received 400 mg/kg of EEMS appeared normal, with no destruction seen. The articular cartilage, fibrous joint capsule and tendon sheath were properly regenerated with no inflammatory cells’ infiltration seen in the synovium.

## Discussion

To our knowledge, this is the first study to confirm the in-vivo anti-inflammatory and anti-arthritic activities of EEMS. Carrageenan-induced edema is a biphasic response with increased vascular permeability occurring between 0 and 2 h following carrageenan injection as a result of histamine, serotonin and kinin release [32]. The late phase, a complement-dependent reaction, indicates the overproduction of prostaglandins and slow reaction substances. Our findings indicated that all three EEMS concentrations (100, 200, 400 mg/kg) produced a dose-dependent activity with the two larger doses (200 and 400 mg/kg) showing statistically significant findings as compared to the standard drug diclofenac. Additionally, EEMS does not confer any apparent acute toxicity when administered up to 2000 mg/kg. Earlier reports on the in vitro anti-inflammatory activity of EEMS leaves [33], as well as the in-vivo anti-inflammatory effects of the ethanolic extract of the leaves [34] or the ethanolic extracts of the stem (20) further supports our results.

CFA-induced arthritis is a systemic disease that manifests as RA-like visceral and articular symptoms. One of the signs of the disease onset is a reduction in the body weight. Animals from the methotrexate-treated and arthritic control groups both had a slight reduction in the body weight, which was nevertheless, ameliorated in the EEMS-treated groups. Paw volume measurement is another important parameter when assessing arthritis [35] since it tends to increase in the arthritis control group, thus confirming the disease onset. Paw volume was dose-dependently reduced following EEMS treatment. SGOT and SGOPT levels indicate liver function. Additionally, as indicators of phagocytic activity, the cellular enzymes can be utilized as sensitive markers of both cellular integrity and toxicity as seen in diseased situations and this is consistent with the lower lysosomal stability seen in adjuvant-induced arthritis [36].

Elevated levels of SGOT and SGPT were observed in the arthritic control group which may be attributed to the injury to the cytoplasm of hepatocytes resulting in a significant increase of SGPT activity [37]. The elevation was ameliorated following treatment with EEMS (400 mg/kg) as also seen with methotrexate [38, 39]. An elevated level of blood urea and serum creatinine was found in the arthritic control group, confirming the presence of some kidney dysfunction as also reported by Ekambaram et al. (2010) [31] and Alamgeer et al., (2017) [40]. Treatment with EEMS and methotrexate however, ameliorated the increased urea and serum creatinine levels.

CRP is a sensitive but non-specific marker of inflammation that responds rapidly to changes in the activity of the underlying inflammatory disease, making its measurement an essential tool in detecting and monitoring inflammatory disease [41]. CRP is an acute-phase protein produced in the liver, mainly by the hepatocytes and blood CRP level correlates well with rheumatoid arthritis (RA) disease severity. Clinical measurement of blood CRP level is indicated for determining disease severity and prognosis for many diseases, including Crohn’s disease, rheumatoid arthritis and even some types of cancer [42]. CRP is a member of the class of acute phase reactants and its level tend to increase dramatically during inflammatory processes [43]. Therefore, as expected, a marked increase in CRP levels was observed in the arthritic control group. Nevertheless, EEMS treatment (400 mg/kg) significantly decreased CRP levels.

ROS production, suggested to mediate cell damage through a number of independent mechanisms including (1) initiation of lipid peroxidation and (2) inactivation of antioxidant enzymes like SOD and GPx and glutathione depletion, is also linked to local and systemic inflammatory responses. Most live cells contain a significant amount of GSH and GSH plays a role in how those cells react to different stimuli. The degree of protection provided by GSH in the reduced state depends on the redox status of the cells [44]. Several diseased animal models have shown decreased GPx activity in tissues under oxidative stress. In order to prevent cell harm, the accumulation of elevated peroxide levels caused by the inactivation of GPx may function as a second messenger and control the production of genes that prevent apoptosis as well as the GPx itself [45]. Hepatic lipid metabolism is linked to oxidative damage in the liver, which may impact the organ’s ability to absorb and transport tocopherol. The morphological damage in the liver occurs before lipid peroxidation and intake of endogenous antioxidants [46].

EEMS decreased the elevated levels of hepatic lipid peroxidation and markedly increased the levels of endogenous antioxidants like SOD, GSH and GPX. *M. serratulum* significantly decreased cathepsin–D activity compared to the arthritic control group. Administration of a high dose (400 mg/kg) EEMS conferred significant cellular and joint structure restoration. Furthermore, oral administration of EEMS (200 and 400 mg/kg) doses significantly reduced the expression of inflammatory marker levels in a dose-dependent manner, as compared to arthritic rats. CFA can alter various gene expressions especially inflammatory and oxidative gene expressions such as iNOS and TNF-α in various tissues [47, 48]. Similarly, the present study indicated that EEMS has good potential anti-arthritic actions occurring via regulation of inflammatory gene expressions. The histopathological findings further corroborated with the biochemical results obtained, thus further confirming that EEMS has a significant protective action against chronic inflammation like arthritis. This potential anti-inflammatory and anti-arthritic properties might be due to the presence of phytochemicals reported earlier in *M. serratulum* [15, 18, 19].

## Conclusions

In the present study, we have confirmed that EEMS significantly reduced chronic inflammation and the paw volume of the affected rats. The suppression of inflammation and arthritic processes is also attributed to their antioxidant activity. Hence, *M. serratulum* is a promising candidate for further preclinical and clinical trials in inflammation and arthritis. However, future research on thorough chemical profiling on EEMS is required to confirm potential components that are responsible for its anti-inflammatory and anti-arthritic properties.

**Fig. 9:**
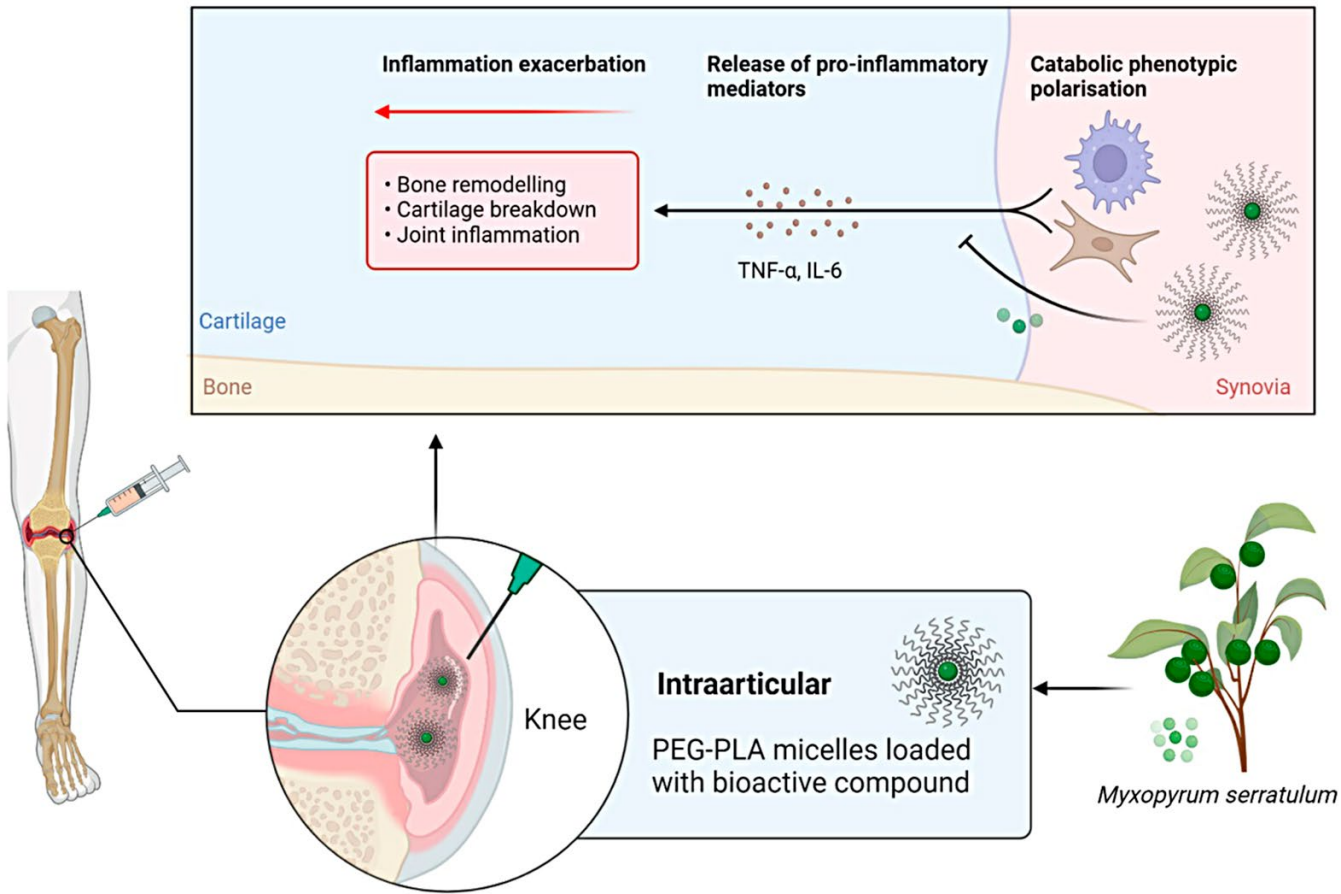
Future perspectives of intra-articular delivery of PEG-PLA micelles-loaded EEMS to be targeted against rheumatoid arthritis (RA). *Abbreviations* *IL-6* Interleukin-6; *TNF-α* Tumour necrosis factor-alpha

In addition, as for the future prospect, EEMS formulation can be improved by using nanoparticles to overcome bioavailability issues [49, 50]. It is recommended to load the extract into polyethylene glycol-polylactic acid (PEG-PLA) micelles to prevent the bioactive component from degradation while enhancing its solubility. Furthermore, when delivered intraarticularly, it induces nanocarriers to accumulate in the target region, allowing better permeation into the cartilage (Fig. 9).

## Data Availability

The datasets used and/or analyzed during the current study are available from the corresponding author on reasonable request.

## Abbreviations

cDNA: Complementary DNA
CFA: Complete Freund’s Adjuvant
CRP: C-Reactive Protein
DMARDs: Disease-Modifying Anti-Rheumatic Drugs
DTNB: Di-thio nitrobenzoic Acid
EEMS: Ethanolic Extract of Myxopyrum serratulum
GADPH: Glyceraldehyde 3-Phosphate Dehydrogenase
GPX: Glutathione Peroxidase
GSH: Glutathione
IL1: Interleukin 1
iNOS: Inducible Nitric Oxide Synthase
MMP: Matrix Metalloproteinase
NSAIDs: Non-Steroidal Anti-Inflammatory Drugs
PPI: Protein-Protein Interaction
RA: Rheumatoid Arthritis
RANKL: Receptor Activator of Nuclear Factor Kappa B Ligand
RNA: Ribonucleic Acid
RT: PCR-Reverse Transcriptase-Polymerase Chain Reaction
SGOT: Serum Glutamic Oxaloacetic Transaminase
SGPT: Serum Glutamic Pyruvic Transaminase
SOD: Superoxide Dismutase
TCA: Trichloroacetic Acid
TNF: Tumor Necrosis Factor
VEGF: Vascular Endothelial Growth Factor
